# Membrane remodeling by the M2 amphipathic helix drives influenza virus membrane scission

**DOI:** 10.1038/srep44695

**Published:** 2017-03-20

**Authors:** Agnieszka Martyna, Basma Bahsoun, Matthew D. Badham, Saipraveen Srinivasan, Mark J. Howard, Jeremy S. Rossman

**Affiliations:** 1 School of Biosciences, University of Kent, Canterbury, Kent, CT2 7NJ, United Kingdom; 2 Department of Cell Biology, University of Texas Southwestern Medical Center, Dallas, Texas, 75390, USA

## Abstract

Membrane scission is a crucial step in all budding processes, from endocytosis to viral budding. Many proteins have been associated with scission, though the underlying molecular details of how scission is accomplished often remain unknown. Here, we investigate the process of M2-mediated membrane scission during the budding of influenza viruses. Residues 50–61 of the viral M2 protein bind membrane and form an amphipathic α-helix (AH). Membrane binding requires hydrophobic interactions with the lipid tails but not charged interactions with the lipid headgroups. Upon binding, the M2AH induces membrane curvature and lipid ordering, constricting and destabilizing the membrane neck, causing scission. We further show that AHs in the cellular proteins Arf1 and Epsin1 behave in a similar manner. Together, they represent a class of membrane-induced AH domains that alter membrane curvature and fluidity, mediating the scission of constricted membrane necks in multiple biological pathways.

Membrane budding is a complex, multi-step process that is involved in many biological processes such as endocytosis, intracellular vesicle trafficking and virus budding. Overall, membrane budding can be divided into four stages: initial deformation of the membrane, formation of a membrane bud, constriction of the membrane neck and membrane scission.

The most studied example of membrane scission is dynamin-dependent scission, which occurs during clathrin-mediated endocytosis. During clathrin-mediated endocytosis, a multitude of proteins are involved in deforming the membrane and forming a membrane bud on the plasma membrane. Dynamin and associated proteins then assemble around the membrane neck, causing a GTP-dependent constriction of the neck that may be sufficient to cause scission^1^,^2^. However, several accessory proteins, including the amphipathic helix (AH) domain-containing proteins, Amphiphysin, Endophilin and Epsin1, have also been suggested to play a role in completing the scission process, following Dynamin-mediated membrane constriction^3^,^4^.

AHs are involved in a wide range of membrane budding events and act by either sensing or generating membrane curvature. Certain AHs such as in the Amphipathic Lipid Packing Sensor (ALPS) motif family, or helices found within N-Bin-Amphiphysin-Rvs (N-BAR)-domain containing proteins, insert into the membrane and sense curvature through hydrophobic interactions^5^,^6^. Curvature-sensing AHs can play essential roles in membrane scission by properly localizing critical components to the membrane neck^5^. In contrast, membrane insertion of cationic AH domains, such as those found in Epsin1 and in antimicrobial peptides, can induce membrane curvature and may play a more direct role in membrane remodeling and scission^7^,^8^. Because of their ability to sense and/or induce membrane curvature, AHs have been implicated in a wide range of membrane scission events, Arf1 for COP-vesicle budding^9^, Endophilin for non-clathrin-mediated endocytosis^10^,^11^, CHMP4b for multi-vesicular body (MVB) budding^12^ and M2 for influenza virus budding^13^. Though, additional proteins are involved in many of these processes and the specific mechanisms of scission often remain unknown.

Influenza virus budding is thought to be initiated by the clustering of large surface-exposed viral glycoproteins in lipid raft domains on the plasma membrane of infected cells^14^. Binding of the matrix protein to the cytoplasmic tails (CT) of the glycoproteins then causes a scaffolding effect, further deforming the membrane and leading to the formation of a membrane bud. The membrane bud is connected to the plasma membrane by a small membrane neck that is constricted by matrix-protein scaffolding on the inside of the bud. It is at this membrane neck that scission occurs, allowing for release of the budding virus^14^,^15^. Recent work has shown that the influenza virus M2 protein, which is not needed for formation of the membrane bud, is essential for completion of membrane scission^13^.

The influenza virus M2 protein is a 97 amino acid homotetrameric protein with a short ecto-domain of unknown function, a transmembrane domain (TMD) and an extended CT that contains a membrane-proximal AH^16^,^17^,^18^. The M2 protein causes alterations of membrane curvature^19^, and the AH domain is essential for membrane scission during virus budding^13^. In addition, expression of the AH alone is sufficient to cause budding and scission in a giant unilamellar vesicle (GUV) system 13, suggesting that the completion of membrane scission is mediated by M2AH. However, similar to cellular AHs, the molecular mechanism by which M2AH causes membrane scission remains unknown. The aim of this study was to determine the molecular mechanism of influenza virus membrane scission and to determine whether this represents a conserved mechanism of cellular membrane scission. Here, we show that hydrophobic membrane interactions drive M2AH domain formation. Insertion of M2AH causes both membrane curvature and ordered lipid domain formation that directly leads to membrane scission. We further show that AHs within Arf1, Epsin1 and possibly EndophilinA3 mediate membrane scission through similar mechanisms. Our results suggest that these AHs are prototypical examples of a protein motif capable of mediating membrane scission.

## Results

### M2AH forms an amphipathic helix upon membrane binding

**Figure 1:**
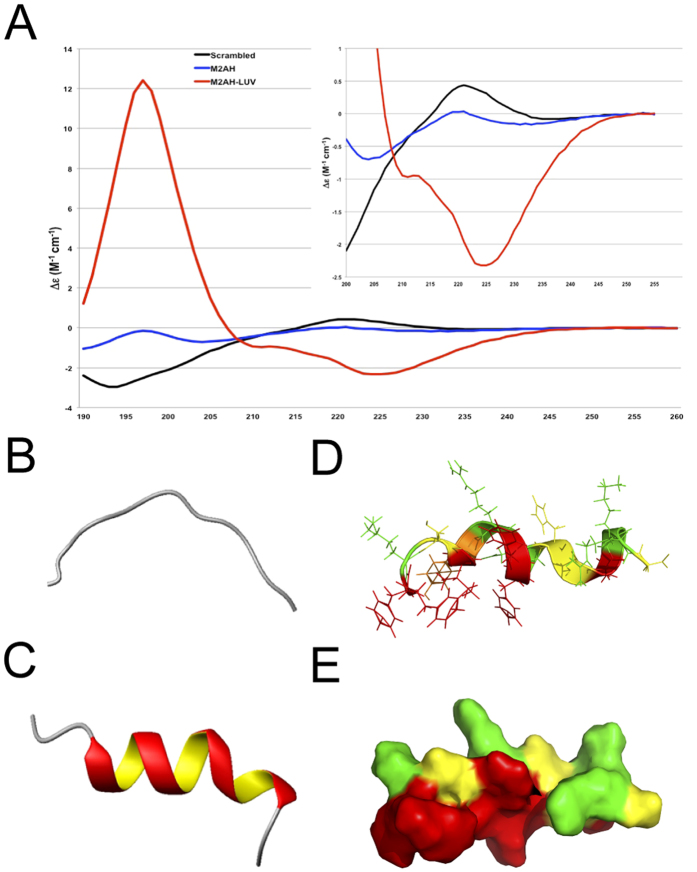
M2AH forms a membrane parallel amphipathic helix upon lipid binding (**A**) SRCD was used to determine peptide secondary structure using a 500 μM solution of the M2AH peptide, a scrambled control peptide solution^13^ or following incubation of 500 μM M2AH peptide with 5 mM POPC:POPG:Ch LUVs. Traces are shown as per-residue molar absorption vs wavelength. Inset shows a zoomed 200–255 nm region of the spectra. (**B**) NMR structure of the M2AH in solution and (**C–E**) in the presence of 100 nm POPC:POPG:Ch LUVs shown as the structure closest to the mean of 20 structure ensembles and represented as a cartoon (**B**,**C**), a ribbon diagram with side-chains (**D**) or a space-filling model. Resides are colored based on hydrophobicity (with hydrophobic side chains in red, neutral in yellow and polar in green). See also Figures S1-S5 and Tables S1–2.

Protein modeling and NMR experiments have suggested that the first 16 amino acids of the A/Udorn/72 influenza virus M2 protein CT (residues 47–62) form an amphipathic helix; however, the structure of the full AH domain and its dependency on lipid binding for **α**-helix formation is not known^16^,^17^,^20^. To determine the secondary structure of the isolated M2AH we used synchrotron radiation circular dichroism (SRCD) spectroscopy to estimate **α**-helix formation. SRCD spectra were obtained for M2AH in detergent-free solution and in the presence of large unilamellar vesicles (LUVs). As shown in Fig. 1a, in the absence of membrane, M2AH is unstructured but rapidly forms an **α**-helix upon membrane binding.

To specifically assess M2AH structure we next determined the high-resolution nuclear magnetic resonance (NMR) structure of the M2AH in solution and bound to LUVs (Fig. 1b–e, S1–4, Tables S1–2). We see that the M2AH is unstructured in solution, with significant movement seen at the N-terminus (Fig. 1b, S2a); however, when bound to LUVs the peptide forms a well-structured, monomeric α-helix from residues 50–61, with a slight kink between residues 58–59 (Fig. 1c–e, S2b). Interestingly, in contrast to bioinformatic predictions, the NMR data show that the AH does not begin until residue 50, with residues 47–49 remaining unstructured. This flexibility may allow for these residues to form the 90 degree turn required to link the TMD to the AH in the full-length protein. Our peptide contains a C50S mutation, used to prevent oxidation; however, in the wild-type protein, C50 is palmitoylated, which may anchor the start of the AH and facilitate its interactions with the membrane.

### M2AH binds membranes through hydrophobic and not charged interactions

**Figure 2:**
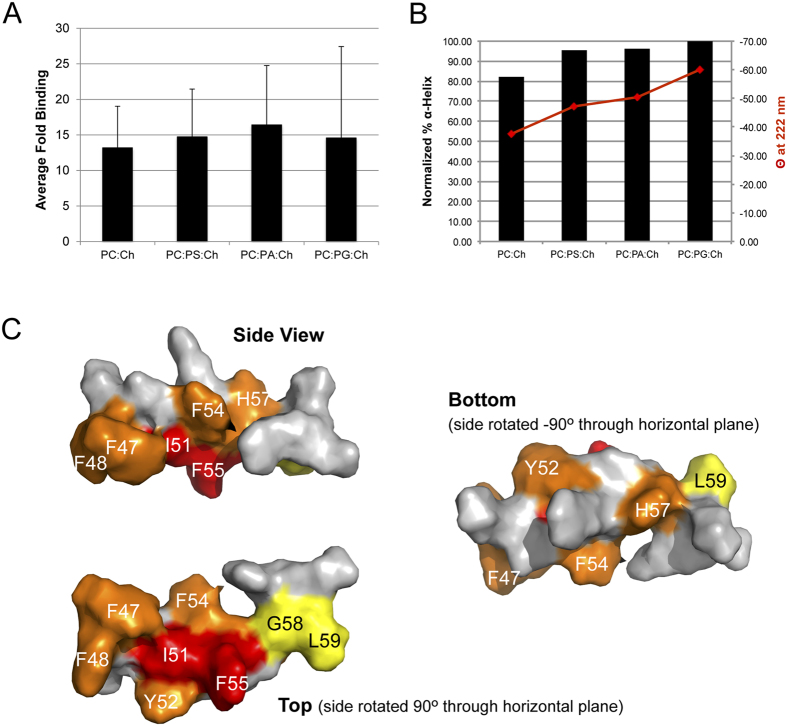
M2AH binds membranes through hydrophobic but not charged interactions. (**A**) 100 μM of FITC-labeled M2AH peptide was incubated with 2.5 mM POPC:Ch LUVs containing 20 molar % of the indicated lipid for 1 hr at room temperature. LUVs were washed and unbound peptide was removed using a 100 kDa filter concentrator. Background binding was determined as above in the absence of LUVs. Fluorescence was determined and is represented as the LUV-bound fluorescence ÷ background-binding fluorescence. Values are the mean ± standard deviation of three independent repeats. No significant differences from POPC:Ch LUVs were detected for any of the lipid combinations using the Student’s T-test (p < 0.05). (**B**) Secondary structure was estimated by CD for 250 μM of the indicated peptide in the presence or absence of 5 mM LUVs. The % α-helix was estimated using K2D3 and normalized to M2AH-bound POPC:POPG:Ch LUVs. (**C**) Space-filling structure of the M2AH with the residues interacting with the membrane colored based on a percentage of the maximum STD transfer (red → blue). See also Figures S6–7 and Table S3.

AH membrane binding can be driven by hydrophobic interactions, polar interactions with lipid headgroups or through a combination of the two. Sequence analysis of the M2AH shows that the polar face possesses several well-conserved cationic residues (K49, R53, K60, R61)^13^, which could interact with anionic lipid headgroups. To test if charge interactions affect M2AH interactions with the membrane, we assessed peptide binding and helical structuring by CD in the presence of charged and uncharged LUVs. To investigate membrane binding by M2AH, we fluorescently labeled a peptide corresponding to M2AH residues 47–62 with FITC, allowed the peptide to bind to LUVs and removed unbound peptide by spin filtration. The fluorescent signal from the LUV-bound M2AH was 5.23 ± 0.91 fold greater than the background, whereas we detected no significant binding of FITC-dextran to liposomes (0.89 ± 0.25 fold increase over background), confirming that M2AH has intrinsic membrane binding capacity. We see that the presence of anionic lipids (PA, PS, PG) in the LUVs did not significantly enhance M2AH binding compared to LUVs containing only PC (Fig. 2a). Similarly, examination of the binding affinity of M2AH for LUVs by Isothermal Titration Calorimetry (ITC) shows comparable binding affinities, with a Kd of 11.4 μM in the presence of PG and 13.4 μM when only PC is present (Figure S5). In addition, increasing the ionic strength of the buffer does not reduce M2AH membrane binding (Figure S6), further suggesting that charge interactions are not essential for M2AH-membrane association. However, CD analysis revealed that the structuring of the M2AH is slightly enhanced in the presence of anionic lipids, increasing α-helix content by an estimated 13% (Fig. 2b). Thus, charge interactions may not be required for M2AH-membrane binding but may facilitate domain formation and stability.

The hydrophobic face of the M2AH contains a significant number of strongly hydrophobic residues, thus, it is possible that M2AH-membrane interactions are primarily driven through hydrophobic interactions. To assess these interactions and determine the key residues involved, we performed saturation transfer difference (STD) NMR. STD results show that many hydrophobic residues interact with the membrane, including: F47, F48, I51, Y52, F54, F55, H57, G58 and L59, though the strongest interactions were seen with: I51, F54, F55 and H57 (Fig. 2c, S7, Table S3). With the exception of H57, these residues mainly group on the hydrophobic face of the helix and suggest that M2AH primarily interacts with the membrane through hydrophobic interactions. However, the identification of H57, suggests that peptide-membrane binding may also be mediated by interactions with the polar lipid headgroups. H57 is seen on the polar face of the amphipathic helix (Fig. 1d) and at the experimental pH (~6.5) may be both protonated and deprotonated. Sequence analysis of other influenza virus strains shows that residue 57 is always either a histidine or another polar/charged amino acid^13^. These results suggest the M2AH interacts with the lipid bilayer through both hydrophobic interactions with the lipid tails and polar interactions with the lipid headgroups.

### M2AH membrane insertion alters membrane order and curvature

**Figure 3:**
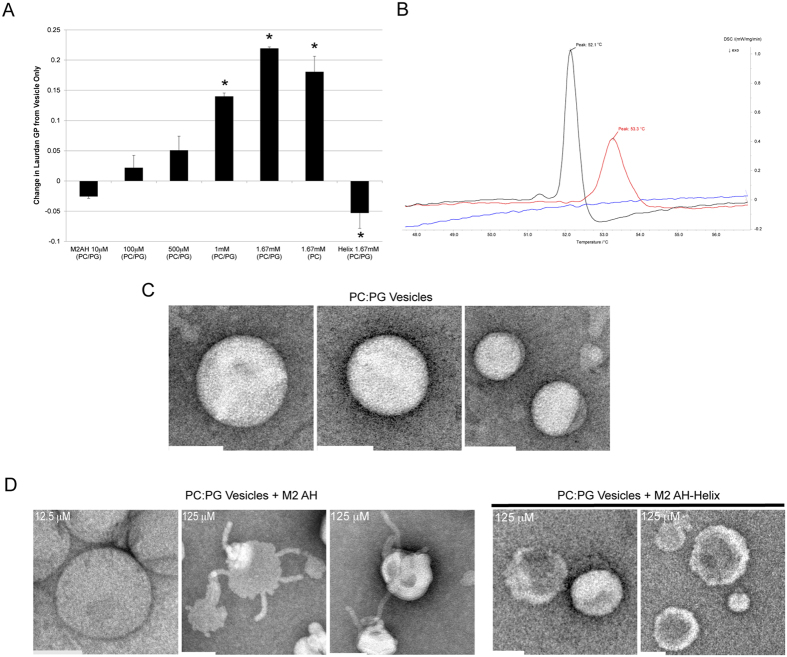
M2AH membrane insertion induces lipid ordering and curvature (**A**) 2.5 mM POPC:POPG:Ch (PC/PG) or POPC:Ch (PC) LUVs were incubated for 1 hr with 25 μM Laurdan dye in the presence or absence of increasing amounts of M2AH peptide or with a single concentration of the control M2AH-Helix peptide. Laurdan fluorescence was then measured at 440 and 490 nm and the ratio was used to calculate the General Polarization (GP) of Laurdan. Values are shown as GP(_LUVs+peptide_) – GP(_LUVs_) and are the mean ± standard deviation of three independent repeats. Significant differences in GP between LUVs + peptide and LUVs were determined by the Student’s T-test (p < 0.05) and are indicated by *. (**B**) DSC curves of 10 mg/ml DSPC MLVs (blue), DSPC MLVs reconstituted with M2AH at a P:L ratio of 1:20 (red) or M2AH alone in solution (black) are plotted as the first differential with the main lipid phase transition temperature noted. (**C**) 5 mM of POPC:POPG:Ch LUVs were incubated for 1 hr with (**D**) 12.5 μM or 125 μM of M2AH peptide or with 125 μM of the control M2AH-Helix peptide before negative staining and TEM visualization. Scale bars indicate 100 nm.

Shallow insertion of an AH into one leaflet of a membrane bilayer can cause the separation of lipid headgroups, creating a lipid packing defect. This effect can be minimized through the induction of membrane curvature^21^. However, deep insertion of an AH into the bilayer, such that the AH resides below the headgroups, minimizes the generation of packing defects. To determine the depth of insertion of M2AH, we reconstituted Laurdan-labeled LUVs in aqueous media and assessed polar headgroup separation. Laurdan is a hydrophobic dye that inserts into the lipid bilayer at the depth of the lipid tails. Laurdan undergoes emission spectrum shifts according to the exposure of the dye to polar media and can be expressed as the general polarization (GP) function^22^. Thus, separation of the lipid headgroups (i.e. the generation of lipid packing defects) would increase media access to the lipid tail-associated dye, changing the emission spectra. Adding M2AH peptide to Laurdan-labeled 100 nm PC:PG LUVs caused a maximum dose-dependent increase in Laurdan GP of + 0.219 ± 0.004 at a P:L ratio of 1:1.5, indicating tighter packing of the surrounding lipids, creating an increase in lipid order which reduces the exposure of the lipid-incorporated dye to the aqueous environment (Fig. 3a). In contrast, addition of the M2AH peptide to Laurdan in solution caused a decrease in Laurdan GP of −0.344 ± 0.133, indicating that the increases seen with M2AH in LUVs is attributable to a change in the hydrophobic environment and not a consequence of direct dye-peptide interactions. Interestingly, the presence of anionic lipids did not affect the ability of M2AH to increase lipid order (Fig. 3a), further supporting the hypothesis that M2AH inserts below the lipid headgroups, into the hydrophobic core of the bilayer, independent of charge interactions. In contrast, the control M2AH-Helix mutant peptide^13^ caused a decrease in Laurdan GP, indicating that the peptide caused a separation of the lipid headgroups, allowing for increased buffer access to the dye (Fig. 3a). This suggests that the mutated peptide, which does not contain any bulky hydrophobic residues, does not insert as deeply into the hydrophobic core of the bilayer as the M2AH peptide.

Next, we analyzed the membrane phase transition temperatures of single-lipid multilamellar vesicles (MLVs) by differential scanning calorimetry (DSC) in order to confirm the effect of M2AH on membrane order. Increased membrane order results in decreased membrane fluidity, which can be detected by DSC as an increase in lipid transition temperature. As it is not possible to clearly resolve the phase transition of lipids with mixed acyl chains (e.g. POPC; 16:0–18:1), we evaluate the effects of M2AH in vesicles made from the related lipid DSPC containing two identical 18:0 acyl chains. We found that the addition of M2AH to MLVs increases the lipid transition temperature of the membrane (Fig. 3b), indicating an increase in lipid order and a decrease in membrane fluidity. This lipid ordering is not generated from existing lipid ordered domains, nor does it require the presence of anionic lipids, but can be created from a membrane consisting of a single lipid species. These results suggest that M2AH increases membrane order through hydrophobic interactions with lipid tails, possibly creating a lipid-ordered domain.

So far we have shown that M2AH forms an amphipathic α-helix upon lipid binding and inserts deep in the membrane through hydrophobic interactions, causing increases in lipid order. To test if M2AH also induces membrane curvature, we incubated LUVs with increasing amounts of M2AH and assessed liposome morphology by Transmission Electron Microscopy (TEM). TEM analysis showed that M2AH alters membrane curvature of 100 nm LUVs, leading to the formation of membrane blebs and short tubes, whereas the control M2AH-Helix peptide did not significantly affect vesicle morphology (Fig. 3c,d).

### M2AH causes membrane scission on pre-constricted membrane necks

**Figure 4:**
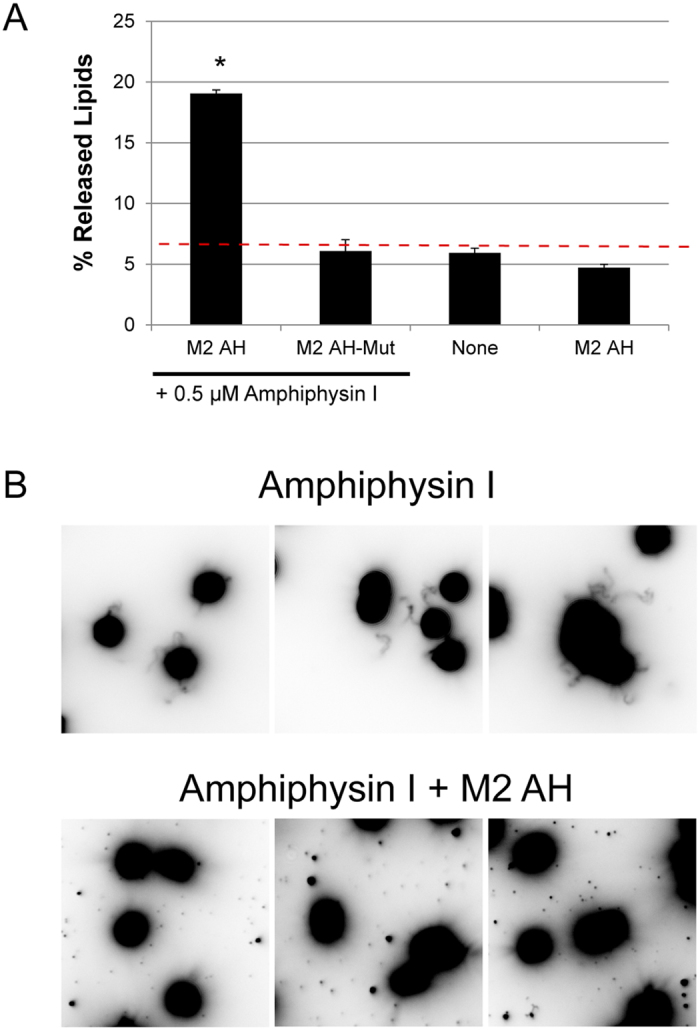
M2AH causes membrane scission on pre-constricted necks. DOPC:DOPS:PIP_2_:RhoPE-containing SUPER templates were tubulated with the addition of 0.5 μM Amphiphysin-I (where indicated) and membrane scission was observed after 30 min incubation in the presence or absence of 25 μM M2AH peptide. (**A**) The resulting supernatant was analyzed for released fluorescent lipid (RhoPE released into the supernatant as % of total RhoPE fluorescence of SUPER templates), shown as mean ± SD for three independent repeats performed in duplicate. Dashed red line indicates the level of background lipid release. Significant difference from background (None: no Amphiphysin-I or peptide) was determined by the Student’s T-test (p < 0.05) and is indicated by *. (**B**) The membrane remodeling events were observed using fluorescence microscopy. Amphiphysin-I causes tubulation of SUPER templates and addition of M2AH leads to release of lipid vesicles (seen as spots). Images are 35 μm square and represented as inverted colors.

Altering membrane fluidity and inducing positive membrane curvature at the center of the constricted neck of a budding virus would cause further constriction of the neck and could lead to membrane scission. To test whether the M2AH causes membrane scission on constricted membrane tubes, we assayed for membrane scission using SUPER Templates^4^. Adding the N-Bar-containing protein, Amphiphysin-I, to the templates caused a scaffolding reaction and the formation of membrane tubes that undergo a background level of spontaneous membrane extraction, unrelated to scission (Fig. 4a, none). The addition of M2AH peptide, but not the M2AH-Helix peptide, to these tubes resulted in significant lipid release, consistent with membrane scission (Fig. 4). Importantly, the M2AH peptide did not cause lipid release in the absence of Amphiphysin-I mediated scaffolding, suggesting that the peptide only acts on specifically scaffolded lipid domains, such as would be seen at the constricted neck of a budding virus (Fig. 4a, M2AH no AmphI).

### M2AH is a canonical member of a family of AH-mediated membrane scission proteins

**Figure 5:**
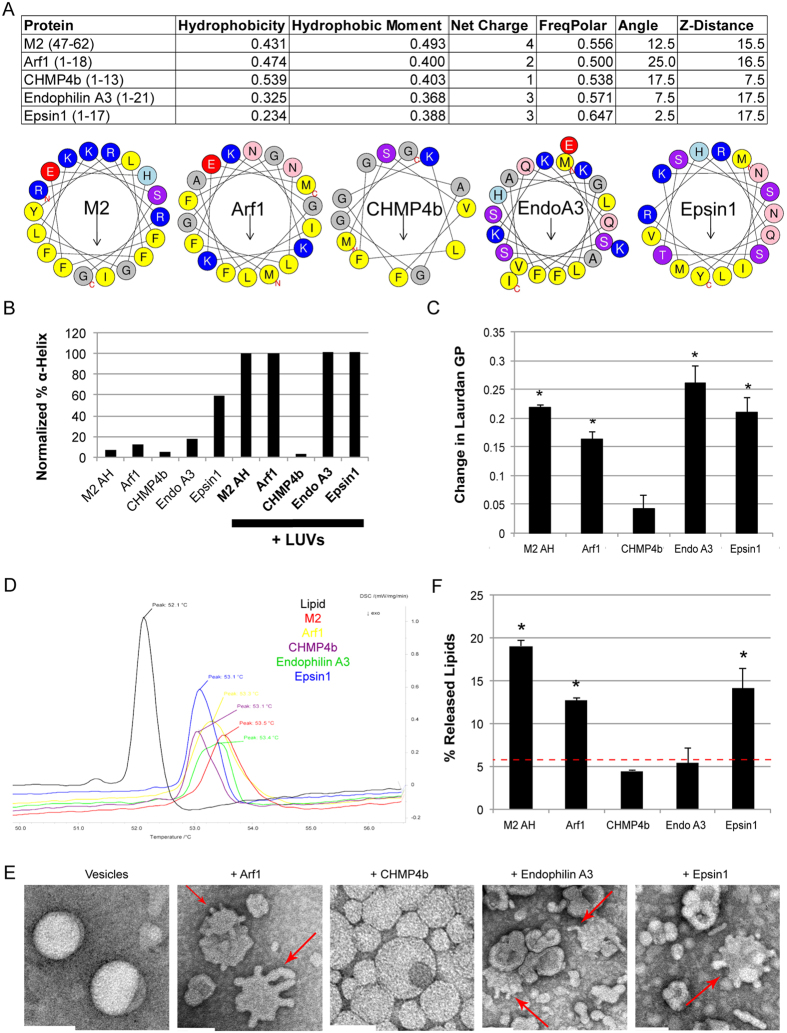
Cellular AHs induce lipid ordering and positive membrane curvature (**A**) Biophysical properties of M2AH and the human Arf1, CHMP4b EndophilinA3 and Epsin1 AHs were calculated using HeliQuest and E(z)3D^53^,^54^. (**B**) Secondary structure was estimated by CD for 250 μM of the indicated peptide in the presence or absence of 5 mM POPC:POPG:Ch LUVs, normalized to M2AH-bound LUVs as in Fig. 2b. (**C**) Changes in Laurdan GP values were calculated from peptide-bound LUVs as in Fig. 3a. Significant difference from LUVs-only was determined by the Student’s T-test (p < 0.05) and is indicated by *. (**D**) DSC curves of DSPC MLVs or DSPC MLVs reconstituted with the indicated peptides was performed as in Fig. 3b. (**E**) TEM analysis of POPC:POPG:Ch LUVs was performed using the indicated peptides as in Fig. 3c,d. Arrows indicate membrane blebs. Scale bars indicate 100 nm. **(F**) DOPC:DOPS:PIP_2_:RhoPE-containing SUPER templates were tubulated with the addition of 0.5 μM Amphiphysin-I and membrane scission was observed after 30 min incubation in the presence or absence of 25 μM of the indicated peptide. The resulting supernatant was analyzed for released fluorescent lipid (RhoPE released into the supernatant as % of total RhoPE fluorescence of SUPER templates, shown as mean ± SD for three independent repeats performed in duplicate). Dashed red line indicates the level of background lipid release. Significant difference from background was determined by the Student’s T-test (p < 0.05) and is indicated by *. See also Figure S8.

M2AH has features in common with cationic antimicrobial curvature-generating helices^5^,^23^. We therefore hypothesized that the M2AH may be part of a larger class of AH domains that mediate membrane scission. Searching for peptides with homology to M2AH did not reveal any close sequence homologues, however, analysis of the AH itself reveals certain identifiable characteristics. Based on bioinformatic analysis, M2AH is 16 amino acids long, has a hydrophobicity of 0.431, a hydrophobic moment of 0.493, a net charge of + 4 and a near-even proportion of polar and hydrophobic residues (Fig. 5a). Using these criteria, we analyzed proteins implicated in cellular budding events and identified similar AHs in Arf1, EndophilinA3, Epsin1 and CHMP4b (Fig. 5a). Previous studies have implicated these AHs in a variety of vesicle budding and scission events^3^,^9^,^10^,^12^. We analyzed the CD spectra of these AHs and found that they are unstructured in solution, with the exception of Epsin1, which is a partially-structured α-helix in solution (Fig. 5b). Upon membrane binding, all peptides except CHMP4b formed α-helices (Fig. 5b).

Interestingly, insertion of Arf1, EndophilinA3 and Epsin1 AHs into a lipid bilayer increased Laurdan GP values (Fig. 5c) and lipid Tm transition temperatures (Fig. 5d), suggesting that these AHs can cause lipid ordering similar to M2AH. Additionally, these AHs enhanced positive membrane curvature, as demonstrated by the formation of membrane blebs and tubules from LUVs (Fig. 5e). CHMP4b did not alter Laurdan GP, slightly affected lipid Tm and did not alter LUV morphology (Fig. 5c–e), consistent with its inability to form an α-helix. To test whether these AHs can also mediate the scission of constricted membrane necks, we assayed for membrane scission using Amphiphysin-I-scaffolded SUPER Templates. Both Arf1 and Epsin1 peptides were able to cause significant vesicle release, similar to the M2AH peptide, whereas scission was not observed with the CHMP4b peptide (Fig. 5f). The EndophilinA3 peptide also did not cause scission in this assay, consistent with previous reports showing that Endophilin-mediated scission is dynamin dependent^24^. These results suggest that AHs of Arf1, EndophilinA3 and Epsin1 may be functionally analogous to M2AH, and together they may represent a family of scission factors.

## Discussion

Many different AHs have been implicated in membrane scission events, though the molecular mechanisms by which these small motifs accomplish scission are often unknown. Here we identify a group of AH-containing scission proteins that appear to function through a similar biophysical mechanism, including Arf1, Epsin1 and the influenza virus M2 protein. The M2AH region is normally unstructured, but rapidly forms a membrane-parallel α-helix upon contact with membranes (Fig. 1). This binding does not require charged interactions and is mediated by hydrophobic interactions between the lipid tails and several bulky hydrophobic residues on one face of the AH (Fig. 2). Upon binding, M2AH inserts deeply into the bilayer, below the lipid head groups, where it induces lipid ordering and possibly the formation of an ordered lipid domain (Fig. 3). Membrane insertion then causes the induction of positive membrane curvature (Fig. 3c,d), leading to scission of constricted membrane necks (Fig. 4).

Influenza virus budding occurs at defined lipid raft domains on the plasma membrane of infected cells^25^. Thus, the budding neck can be thought of as the lipid phase boundary between the viral membrane and the bulk plasma membrane. As such, lipid phase boundaries are under considerable strain, and lipid phase segregation can be sufficient to cause budding in artificial membrane systems, though it is unclear if phase segregation is sufficient to cause budding *in vivo*^26^,^27^,^28^. Thus, the membrane neck shows existing constriction and strain arising from the scaffolding and lipid phase segregation that occur as a part of the budding process. This results in a strained and constricted neck with a diameter of 25.97 ± 11.25 nm (calculated from TEM images of stalled buds formed during a ΔM2 influenza virus infection)^13^. However, the scaffolding and phase segregation alone are not sufficient to cause membrane scission in the absence of M2AH^13^, nor is the M2AH capable of causing membrane scission in the absence of a constricted neck (Fig. 4, compare M2AH with M2AH no AmphI).

A constricted neck is shaped as a catenoid and has negative Gaussian curvature (NGC) that can be quantified as the variable K. It has been previously shown that the M2 protein can induce NGC in membranes of *K = *−*0.0403 nm*^−2 19^. Given that *K(z) *=* *−*sech(z/c)*^4^*/c*^*2*^, where *2c* is the diameter of the midpoint of the neck at *z = 0*, it is possible to determine the NGC of a constricted membrane neck by solving for *K = *−*1/c*^*2*^. The ΔM2 virus has a membrane neck of *25.97 nm*, giving *K = −0.00593 nm*^*−2*^. Adding the K values for the ΔM2 membrane neck with that of the M2 protein gives *K = −0.04623 nm*^−*2*^, yielding a new membrane neck diameter of *4.67 nm*. This calculation assumes similar bending rigidity between the experimental SUVs used by Schmidt *et al*. and the membrane neck of a budding virus, as such it represents only an estimation of the constricting capacity of the M2 protein. However, this suggests that M2AH-induction of membrane curvature on pre-constricted necks may be sufficient to reduce the neck diameter to below 5 nm. It has been estimated that spontaneous membrane scission can occur when the bilayer is constricted below 5 nm^29^. Thus, the combination of scaffolding-induced constriction, coupled with curvature induction by the M2 protein may be sufficient to cause membrane scission. Furthermore, this calculation may underestimate the amount of curvature generated by the M2 protein *in vivo*. Insertion of M2AH into a bilayer causes increases in lipid order (Fig. 3a,b). Several proteins that mediate the analogous process of membrane fusion have also been found to induce lipid ordering and membrane strain, a property thought to significantly enhance fusion^30^,^31^,^32^. Increasing lipid order at the neck would put further strain on the constricted, phase-separated membrane neck, enabling additional constriction and causing membrane scission (Fig. 4). Thus, M2AH causes membrane scission by exploiting the underlying biophysical properties of lipid bilayers.

Our data show that AHs from Arf1 and Epsin1 may also function in a similar manner to M2AH (Fig. 5), suggesting that influenza virus budding, endocytosis and COP vesicle budding may share a similar mechanistic basis for the completion of membrane scission. Both Arf1 and Epsin1 have already been shown to play important roles in membrane scission during COP vesicle budding^9^, actin-mediated endocytosis^33^ and clathrin-mediated endocytosis^3^, though Dynamin is also essential for the completion of clathrin-mediated endocytosis^34^. The Epsin1 AH is located within the larger Epsin N-terminal homology (ENTH) domain, which can tubulate liposomes^7^, similar to the full-length Arf1 protein^35^ and M2AH (Fig. 5c). Interestingly, membrane insertion of the Arf1 and Epsin1 AHs is specifically regulated by GTP binding^35^,^36^ and PI_(4,5)_P_2_ binding, respectively^37^ in the context of the full-length proteins; however, when expressed alone, the Arf1 and Epsin1 AH domains can insert into membranes in the absence of their specific triggers (Fig. 5). This suggests that the underlying biophysical mechanism by which these AHs facilitate membrane scission is similar, though during *in vivo* budding their activity may be subjected to more complex regulation.

Endophilins contain AHs within N-BAR domains, cause tubulation of liposomes and have recently been shown to be involved in membrane scission during clathrin-independent endocytosis^3^,^10^,^11^,^38^. Our results show that whilst EnodphilinA3 is capable of altering membrane curvature and fluidity it does not cause scission in the specific conditions tested, consistent with the reported dependency on dynamin for scission^24^. This suggests that N-BAR-domain containing proteins such as EndophilinA3 and Amphiphysin-I may scaffold and constrict budding necks, facilitating, rather than causing, membrane scission (Fig. 5). Similarly, CHMP4b is essential for ESCRT-dependent MVB budding and scission^12^, though the protein does not cause liposome tubulation. It is thought that the AH functions as a protein anchor and curvature sensor rather than as a membrane deformer^39^. These results are consistent with our observations that CHMP4b does not form a membrane scission-competent AH (Fig. 5); thus, ESCRT-mediated membrane scission may proceed through a fundamentally different mechanism. However, just as with influenza virus budding, ESCRT-mediated membrane scission and COP budding appear to occur at membrane phase boundaries and may involve the modulation of line tension by the formation of specific lipid domains^40^,^41^. Thus, we speculate that membrane scission may require several alterations of membrane properties, including: the induction of positive curvature, the modification of existing line tension and the creation of new lipid ordering. Each of these modifications would further constrict a pre-constricted neck while placing the membrane under increasing strain, enabling spontaneous membrane scission to occur.

We propose that the M2AH domain is a canonical member of a class of AH domains that mediate membrane scission by modifying basic membrane properties. We suggest that the AHs within Arf1, Epsin1 and possibly EndophilinA3 are members of this novel family, and anticipate that future research will identify homologous domains in many other proteins that regulate membrane scission in a wide variety of essential budding processes.

## Materials and Methods

All peptides were synthesized by Biomatik (DE, USA) at 98% purity, with TFA removed. Peptide sequences included N-terminal acylation and C-terminal amidation where indicated. Sequences were: M2AH (47–62 of A/Udorn/72) Ac-FFKSITRFFEHGLKRG-Am; M2AH-Scrambled (randomized version of the M2AH)^13^; Arf1 (1–18) MGNIFANLFKGLFGKKEM-Am; CHMP4b (1–13) MSVFGKLFGAGGG-Am; Endophilin A3 (1–21) MSVAGLKKQFHKASQL FSEKI-Am; Epsin1 (1–17) MSTSSLRRQMKNIVHNY-Am. Fluorescent M2AH peptide incorporates FITC conjugated to K60. Lipids were dissolved in chloroform and include: Cholesterol (Ch), 1,2-dioleoyl-sn-glycero-3-phosphocholine (DOPC), 1,2-dioleoyl-sn-glycero-3-phospho-L-serine (DOPS), L-α-phosphatidylinositol-4,5-bisphosphate (PI_(4,5)_P_2_), 1,2-dioleoyl-sn-glycero-3-phosphoethanolamine-N-(lissamine rhodamine B sulfonyl) (RhPE), 1,2-distearoyl-sn-glycero-3-phosphocholine (DSPC), 1-palmitoyl-2-oleoyl-sn-glycero-3-phosphate (POPA), 1-palmitoyl-2-oleoyl-sn-glycero-3-phosphocholine (POPC), 1-palmitoyl-2-oleoyl-sn-glycero-3-phospho-1-rac-glycerol (POPG), 1-palmitoyl-2-oleoyl-sn-glycero-3-phospho-L-serine (POPS) (Avanti Polar Lipids, AL, USA).

### Large Unilamellar Vesicles

LUVs were made by extrusion. Lipid solutions containing 12.5 μmole of lipids were made using a 4:1:0.025 molar ratio of POPC:POPG:Ch or a 5:0.025 molar ratio of POPC:Ch, expect where indicated and as previously described^13^. In brief, the lipid solution was dried under a stream of argon gas, residual chloroform was then removed by storage for 1 hr under vacuum. Lipid films were then dissolved in 500 μl of potassium buffer (10 mM K_2_HPO_4_, 50 mM K_2_SO_4_, 5 mM MOPS, pH 7.4) and hydrated for 30 min at 5 °C above the phase transition temperature of the lipid mix with vortex mixing every 5 min. Lipid solutions were then freeze-thawed 15 times transferring between a dry ice ethanol bath and a water bath at hydration temperatures before extrusion using an Avanti Mini-Extruder, 26 times with 100 nm pore-size membranes (Whatman Nuclepore Track-Etched Membranes, GE Healthcare Bio-Sciences, PA, USA) at hydration temperatures. LUVs were stored at 4 °C and used within one week.

### Dynamic Light Scattering

Vesicle size was measured using the Nano-ZS Zetasizer (Malvern Instruments, Malvern, UK). 4 μl of vesicles were mixed with 32 μl of water, measurements were made using Zen40 micro cuvettes (Malvern Instruments) at 25 °C.

### Circular Dichroism

CD experiments were performed using both the nitrogen-flushed Module B end-station spectrophotometer at B23 Synchrotron Radiation Circular Dichroism (SRCD) Beamline at the Diamond Light Source^42^,^43^,^44^ and a JASCO J-715 spectropolarimeter (Jasco, MD, USA). For SRCD measurements, the M2AH peptide or a scrambled control peptide was diluted in water to a final concentration of 500 μM. Where indicated the M2AH peptide was mixed with a 5 mM solution of 100 nm POPC:POPG:Cholesterol LUVs (4:1:0.025 molar ratio), at a final concentration of 500 μM. SRCD spectra were acquired using an integration time of 2 s, a demountable cell of 0.005 cm pathlength and 1.2 nm bandwidth at 20 °C. Spectra were normalized using average amino acid molecular weight of 113 and the results processed using CDApps^45^ and OriginLab (Origin Labs, MA, USA). Secondary structure estimation (SSE) from CD spectra was carried out using CDApps using Continll algorithm^46^.

For measurements on the JASCO J-715, 5 mM of lipid vesicles were mixed with 250 μM of the indicated peptide and diluted with water to a final volume of 300 μl. Blank readings were made using water or 5 mM lipid vesicles in water, for measurement of the peptide in the solution and the peptide with lipids respectively. All measurements were done in quadruplicate using 1mm path length glass cuvettes (Starna Scientific, Essex, UK) at 25 **°**C. Respective blank measurements were subtracted from all samples and the percentage of α-helix was calculated using K2D3 at http://k2d3.ogic.ca/47.

### Nuclear Magnetic Resonance Spectroscopy

M2AH structures were examined using a 500 μM solution of the 16aa A/Udorn/72 M2AH domain in 90% H_2_O/10% D_2_O with 10 μM POPC:POPG:Cholesterol LUVs, where indicated. Samples were placed in 3mm glass tubes (Shigemi, PA, USA) and data was collected using a 14.1 T (600 MHz ^1^H) Bruker (MA, USA) Avance III NMR spectrometer with a QCI-F cryoprobe at 25 °C. Total correlation spectroscopy (TOCSY) and Nuclear Overhouser effect spectroscopy (NOESY) data sets were obtained in a two-dimensional (2D) NMR experiment with assignments generated using CcpNmr Analysis^48^ and structural ensembles calculated using CNS^49^. ^1^H chemical shifts and through-space structural assignments were acquired from TOCSY and NOESY data sets. Dihedral angles were estimated using the DANGLE module of CcpNmr Analysis^50^. Hydrogen bonds were estimated using MOLMOL^51^ by analysing the calculated structure and distance between oxygen and nitrogen-bound hydrogen atoms and were added for the pairs of atoms that were less than 3 Å apart. The final ensemble was energy minimised in a water environment using YASARA (YASARA Biosciences, Vienna, Austria). The precision and quality of the calculated structures was checked by Ramachandran analysis using PROCHECK^52^.

STD-NMR studies were performed as above with 3-9-19 WATERGATE and Gaussian STD excitation pulses of 20ms duration and a γB_1_ of 140 Hz that were applied for 2 s at −3ppm and −30ppm for saturation and control respectively. ^1^H T_1_ relaxation time constants were obtained using an inversion-recovery sequence, including 3-9-19 WATERGATE. To obtain T_1_ inversion-recovery 180⁰-τ-90⁰delays in 0.1 s steps between 0.2 and 0.1 s were used. Intensities for STD and T_1_ relaxation experiments were obtained using Spectrus Processor (ACD Laboratories, Ontario, Canada).

### Isothermal Titration Calorimetry

ITC was performed on a MicroCal VP-ITC (Malvern) with the cell filled with 50 μM M2AH peptide, buffer matched to the LUVs, and 20 × 13 μl injections of 5 mM lipid vesicles. Peak integration, curve fitting and data analysis was performed using MicroCal Origin software (Malvern) as per the manufacturers recommendations.

### Peptide Binding Assay

100 μM of M2AH FITC labeled peptide was mixed with 2.5 mM of the indicated lipid vesicles and diluted with water to a final volume of 50 μl. Controls were made with 100 μM of M2AH FITC labeled peptide and water. Samples were incubated for 1 hr at room temperature in the dark to allow for peptide-membrane binding. After incubation, unbound peptide was removed by washing twice through 100 kDa Amicon Ultra centrifugal filters (Merck Millipore, Watford, UK). Retained fluorescence was determined on the FLUOstart Omega fluoresecent plate reader (BMG Labtech, Bucks, UK) using a 492 nm excitation filter and a 520 nm band pass emission filter. All data was blank corrected before processing and all experiments were performed in triplicate.

### Laurdan Assay

2.5 mM of lipid vesicles were mixed with 25 μM Laurdan dye (Life Technologies, Paisley, UK) and 200 μM of peptide, where indicated, in a total volume of 50 μl. Fluorescence was measured on a Cary Eclipse fluorescence spectrophotometer (Agilent Technologies, CA, USA) using a 355 nm excitation filter recording fluorescence emissions at 440 and 490 nm. All experiments were performed in triplicate. Laurdan GP was calculated using equation: GP = (I_440_−I_490_)/(I_440_ + I_490_).

### Electron Microscopy

For analysis of LUVs, 5 mM solutions of lipid vesicles were incubated with 125 μM of the indicated peptide for 1 hr at room temperature and stained with uranyl acetate before imaging. Samples were imaged on a JEOL 1230 (Tokyo, Japan) electron microscope using a Gatan digital camera (CA, USA). Post image processing was limited to cropping and equal adjustment of image levels.

### Differential Scanning Calorimetry

Multilamellar vesicles (MLVs) were made by drying 13 mmole of DSPC lipid under a stream of argon gas, followed by incubation under vacuum for 1 hr to remove residual chloroform. Lipid films were then reconstituted in 1 mM Hepes buffer (pH 7.2) containing 650 μM of the indicated peptide, giving a 10 mg/ml lipid solution and a peptide : lipid ratio of 1:20. MLVs were formed following 30 min of hydration at 60 °C with regular vortexing. Samples were then analyzed on a Phox 200PC DSC (Netzsch, Germany) using sealed aluminum crucibles and under N_2_ gas. All runs were corrected to a baseline run using empty crucibles. Samples were scanned from 35 °C − 75 °C at a rate of 10 K/min with 2 min isothermal holds at the end of each run. All samples were scanned four times, with the first scan discarded. Subsequent scans were compared to ensure consistency in the identified lipid Tm. After each sample run, crucibles were weighed to ensure no weight loss, and thus no sample loss, had occurred. Netzsch Proteus Analysis software was used to graph the first differential of the data and to identify Tm peaks through the automatic peak calling function near the 55 °C Tm of DSPC.

### SUPER Templates

The SUPER template assay for membrane fission was performed as previously described^4^ with few modifications. Briefly, 100 nm liposomes (DOPC:DOPS:PIP_2_:RhPE at a molar ratio of 79:15:5:1) were prepared in water and incubated with 2.5 μm silicon microspheres in 350 mM NaCl. Extensively washed, lipid coated beads were then incubated with 0.5 μM Amphiphysin and/or 25 μM M2AH, where indicated, (in 20 mM Hepes, 150 mM KCl, pH 7.5) for 30 min and the supernatant was observed for released vesicles via either fluorescence measurement in a plate reader setup (compared to total lipids determined by dissolving the SUPER Templates in 0.1% Triton X-100) or visualization using an inverted Olympus IX-70 microscope with a 100X, 1.35 NA oil-immersion objective. Data are the average of 3 independent trails, each performed in duplicate.

## Additional Information

**How to cite this article:** Martyna, A. *et al*. Membrane remodeling by the M2 amphipathic helix drives influenza virus membrane scission. *Sci. Rep.* **7**, 44695; doi: 10.1038/srep44695 (2017).

**Publisher's note:** Springer Nature remains neutral with regard to jurisdictional claims in published maps and institutional affiliations.

## Supplementary Material

Supplementary Information
