# Supplementary material for: Membrane remodeling by the M2 amphipathic helix drives influenza virus membrane scission
Source: Sci Rep. 2017 Mar 20;7:44695. doi: 10.1038/srep44695 (PMC5357790; doi:10.1038/srep44695)
Supplement: Supplementary Information [file srep44695-s1.pdf]

Membrane remodeling by the M2 amphipathic helix drives influenza virus membrane scission

Agnieszka Martyna[1], Basma Bahsoun[1], Matthew D. Badham[1], Saipraveen Srinivasan[2], Mark J. Howard[1,§] and Jeremy S. Rossman[1,*]

[1]School of Biosciences, University of Kent, Canterbury, Kent, CT2 7NJ, United Kingdom

[2]Department of Cell Biology, University of Texas Southwestern Medical Center, Dallas, Texas, 75390, USA

[§]Present address: Centre for Microscopy, Characterisation and Analysis, University of Western Australia, Perth, WA 6009, Australia

*To whom correspondence should be addressed: Jeremy S. Rossman, School of Biosciences, University of Kent, Canterbury, Kent, CT2 7NJ, United Kingdom.

Telephone: +44 (0)1227823207; Fax: +44 (0)1227763912; e-mail: j.s.rossman@kent.ac.uk

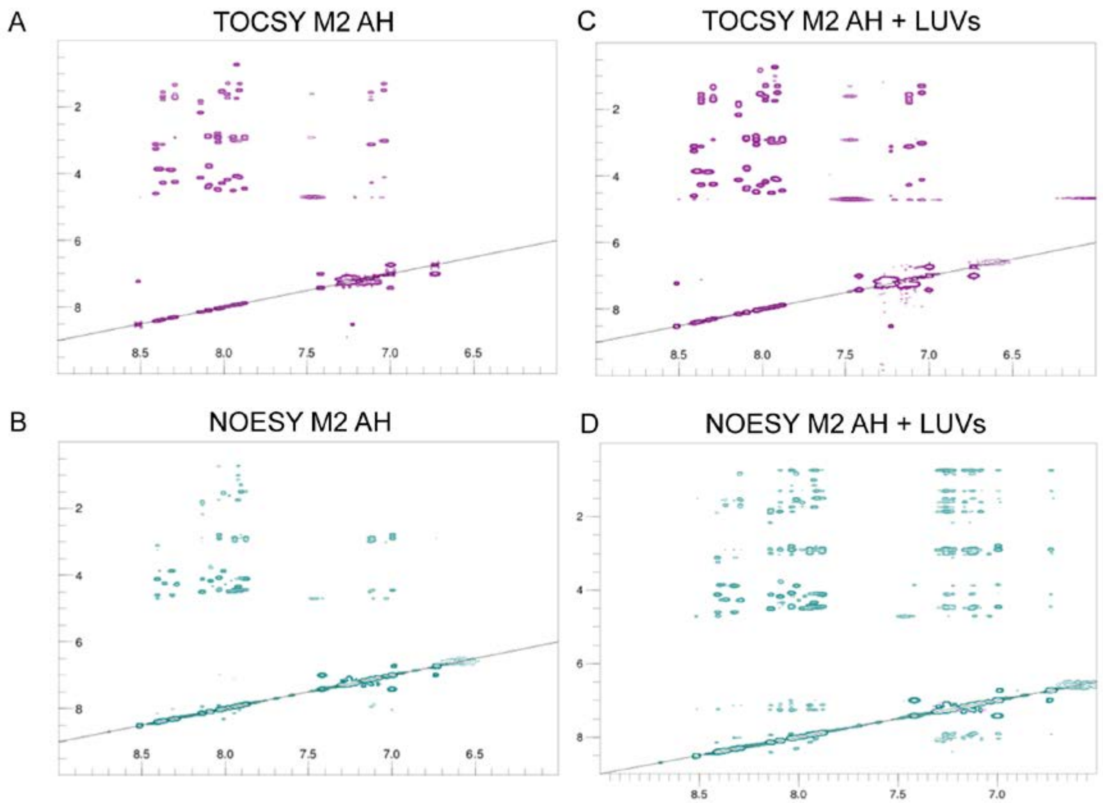

Figure S1. M2AH NMR spectra.

A, C) TOCSY and (B, D) NOESY NMR spectra of the fingerprint region of M2AH in solution (A-B) or in the presence of 100nm POPC:POPG:Cholesterol LUVs (C-D).

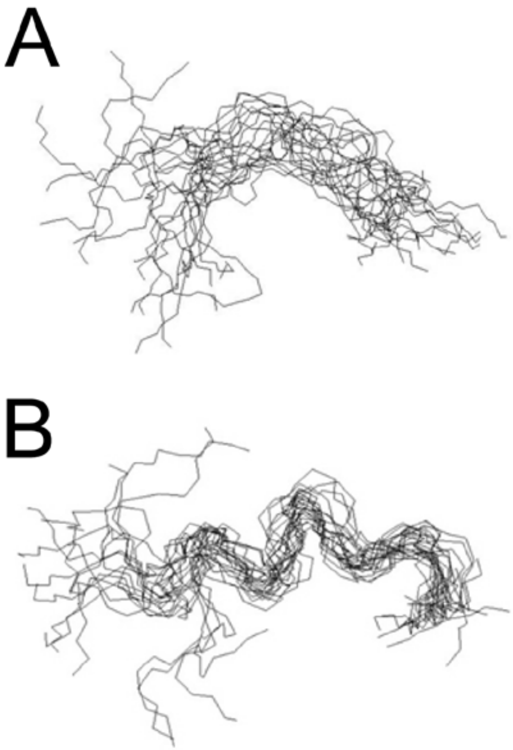

Figure S2. M2AH NMR structures.

A) Models of the M2 AH peptide in solution and (B) in the presence of LUVs were composed of 20 structure ensembles fitted over the backbone atoms in residues 4-16. Calculated structures show random coil confirmation for the M2 AH peptide in solution and formation of an α-helix between residues 4-15 (residues 50-61 of a full length M2 protein) of the peptide in the presence of LUVs.

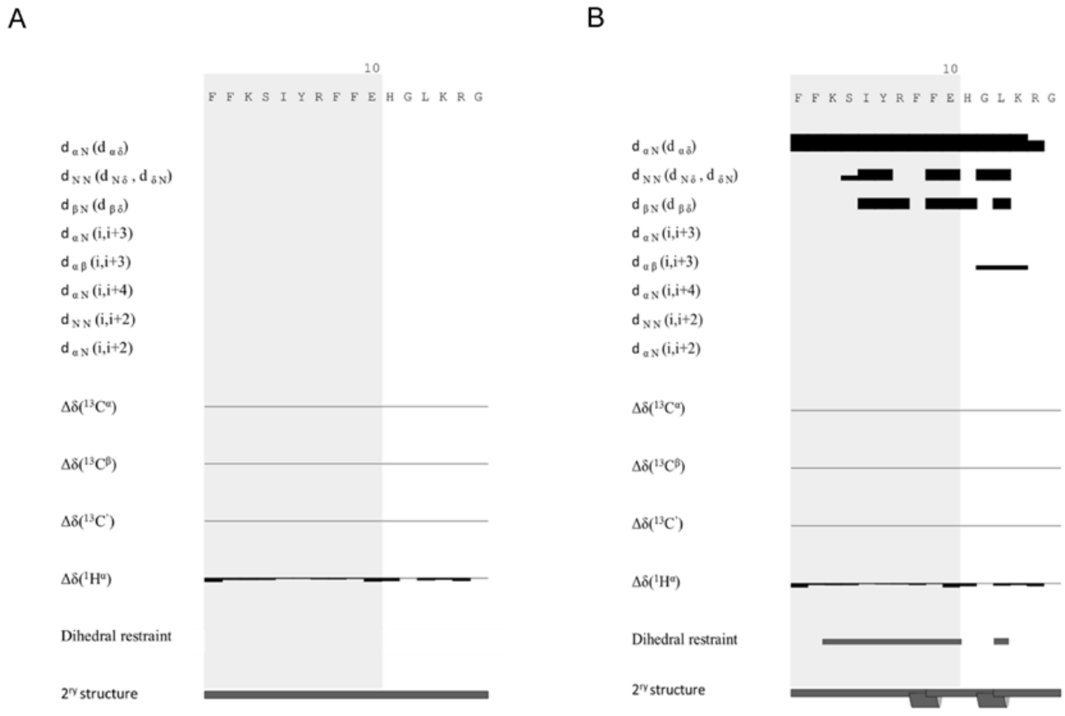

Figure S3. NOE contacts for M2AH.

A) NOE contacts, chemical shift difference, hydrogen bond donors and dihedral restrains for M2AH in solution or (B) in the presence of 100nm POPC:POPG:Cholesterol LUVs. The secondary structure prediction indicates the limits of Ramachandran analysis for small peptides.

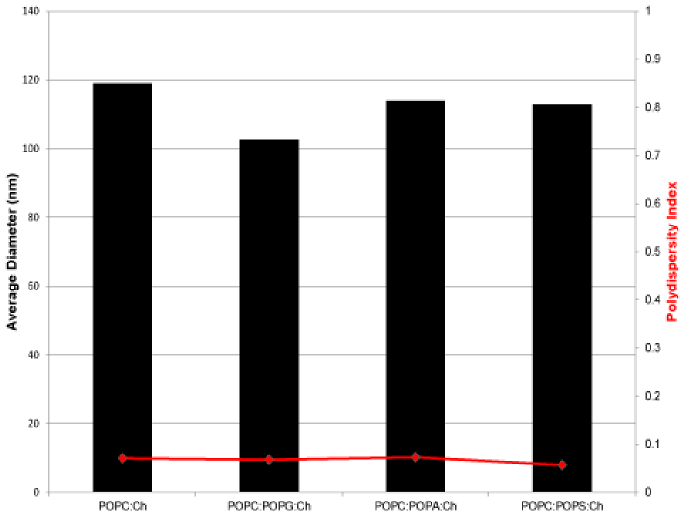

Figure S4. Liposome size profiles.

The average diameter (in nm) and polydispersity index of all SUV and LUVs was determined by DLS at 25^{0}C.

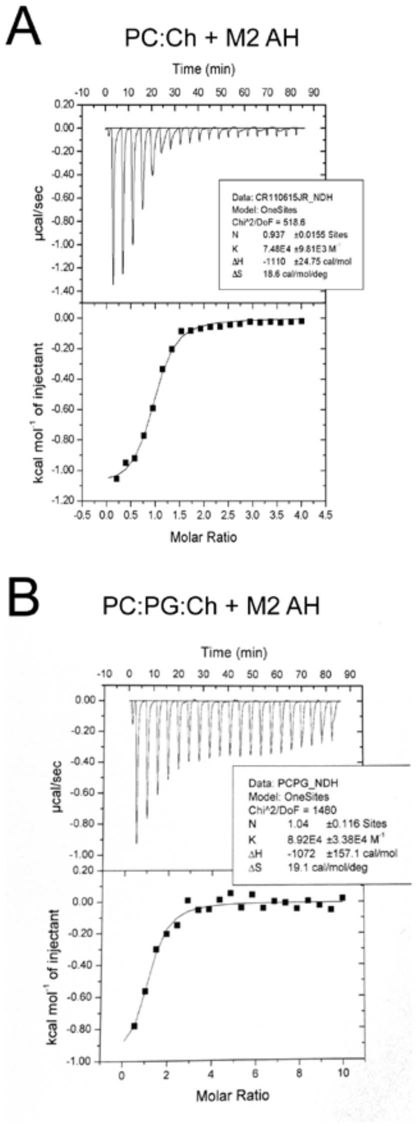

Figure S5. Membrane binding affinity of M2AH.

A) ITC profiles were determined following 20 x 13µl injections of 5mM 100nm POPC:Ch or (B) POPC:POPG:Ch LUVs into a 50µM M2AH peptide solution.

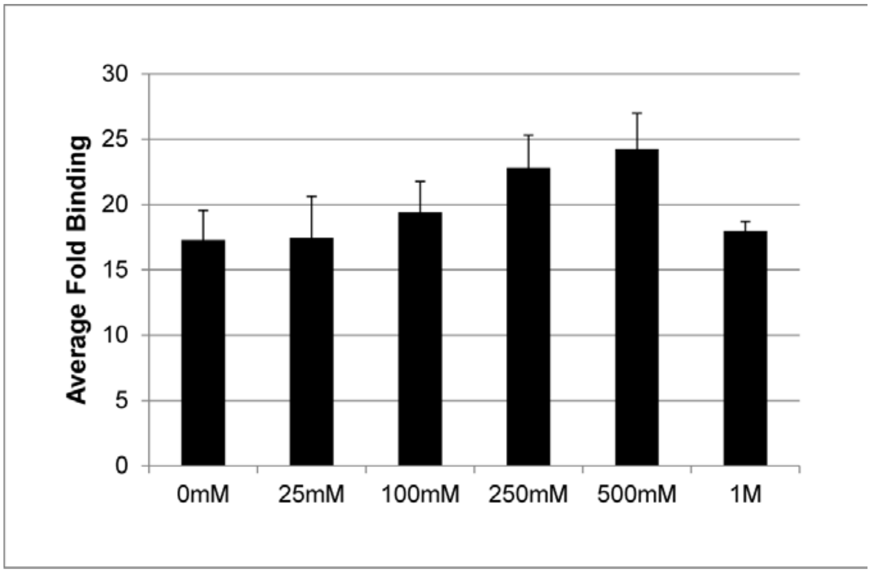

Figure S6. M2AH-membrane binding is not charge-dependent.

FITC-labeled peptide binding was determined as in Figure 2e using LUVs made in 1mM Hepes buffer (pH 7.2) containing POPC:POPG:Ch with increasing amounts of NaCl to reach the indicated total ionic strength of the buffer.

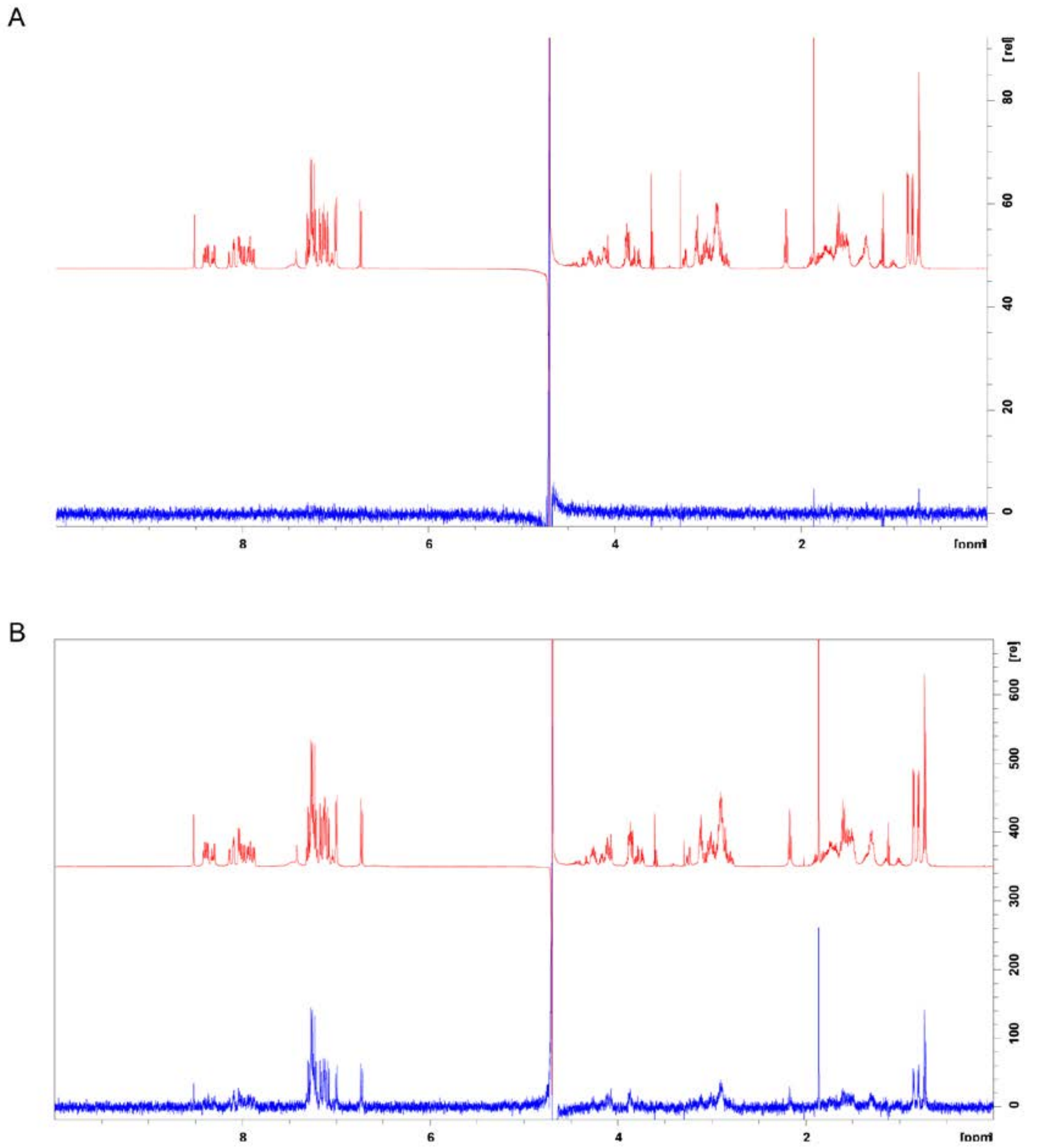

Figure S7. STD NMR spectra.

One-dimensional STD NMR was used to determine the interaction between M2AH and the lipid membrane. A) STD control (red) and difference spectra (blue) for M2AH in solution and (B) in the presence of 100nm POPC:POPG:Cholesterol LUVs.

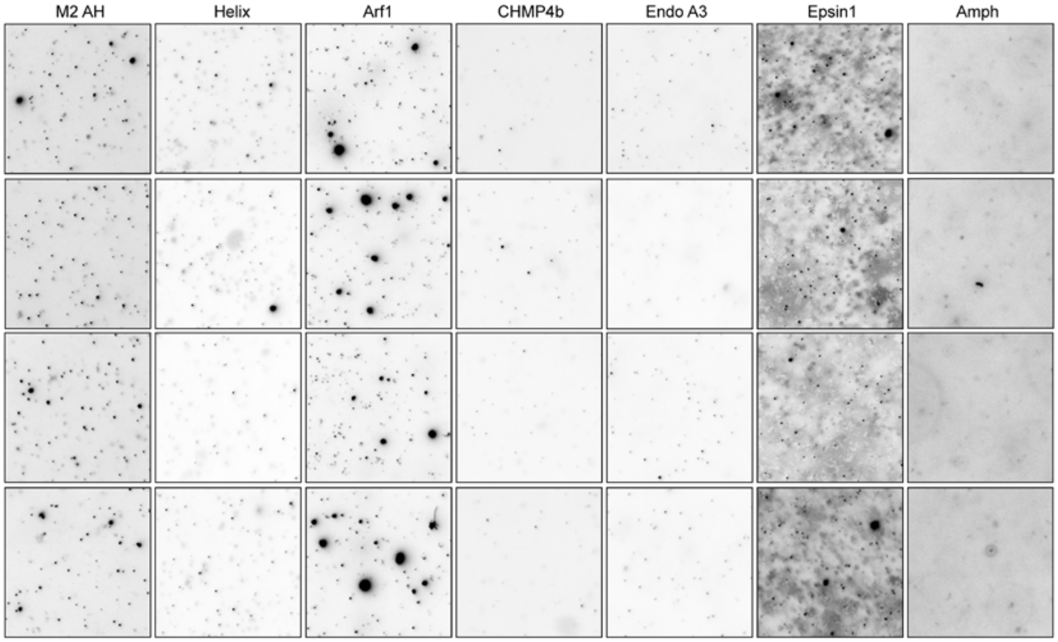

Figure S8. Cellular AHs cause scission of amphiphysin-tubulated SUPER templates.

Pre-formed RhoPE labeled SUPER templates were mixed with 0.5 μM Amphiphysin-I and 25 μM of the indicated peptide for 30 min at RT and resulting membrane remodeling events were observed using fluorescence microscopy. Images are 35 μm square and represented as inverted colors.

Supplemental Table 1

Residue	H^N	H^α	H^β	H^γ	H^δ	H^ε
47 Phe	-	4.41	2.86			
48 Phe	8.03	4.49	2.91 3.05			
49 Lys	7.98	4.17	1.73	1.29	1.61	
50 Ser	8.09	4.34	3.76			
51 Ile	7.92	4.08	1.74		0.73	
52 Tyr	8.04	4.45	2.80 2.91			
53 Arg	7.91	4.11	1.50	1.30	3.02	7.04
54 Phe	7.88	4.44	2.88 2.95			
55 Phe	7.94	4.51	2.89 2.99		7.12	
56 Glu	8.14	4.12	1.82 1.90	2.17		
57 His	8.41	4.59	3.11 3.24			
58 Gly	8.32	3.88				
59 Leu	8.01	4.28	1.53		1.53	
60 Lys	8.3	4.25	1.68 1.74	1.35	1.60	2.91
61 Arg	8.37	4.27	1.69 1.79	1.56	3.12	7.11
62 Gly	8.39	3.86				

Table 1a. Chemical shifts (ppm) of the M2AH in solution.

Residue	H^N	H^α	H^β	H^γ	H^δ	H^ε
47 Phe	-	4.41	2.86		7.09	7.16
48 Phe	8.04	4.49	2.90 3.04		7.16	7.27
49 Lys	7.98	4.17	1.73	1.29	1.61	2.92
50 Ser	8.09	4.34	3.77			
51 Ile	7.92	4.08	1.74	1.15 1.00	0.73	
52 Tyr	8.04	4.45	2.90 2.79		6.99	7.08
53 Arg	7.91	4.11	1.5	1.30	3.02	7.04
54 Phe	7.88	4.44	2.88 2.96		7.12	7.26
55 Phe	7.95	4.5	2.89 2.99		7.13	7.26
56 Glu	8.14	4.12	1.81 1.89	2.16		
57 His	8.41	4.6	3.11 3.26		7.23	
58 Gly	8.33	3.88				
59 Leu	8.01	4.28	1.53		0.83	
60 Lys	8.29	4.24	1.74 1.69	1.35	1.61	2.91
61 Arg	8.37	4.27	1.69 1.79	1.55	3.12	7.12
62 Gly	8.39	3.86				

Table 1b. Chemical shifts (ppm) of the M2AH in presence of 100nm POPC:POPG:Cholesterol LUVs.

Supplemental Table 2

	In solution	In presence of LUVs
**NMR distance and dihedral constrains**		
Distance constraints		
Total NOE	38	218
Intra-residue	3	55
Inter-residue	35	163
Sequential ($\|i - j\| = 1$)	35	91
Medium-range ($\|i - j\| < 4$)	35	160
Long-range ($\|i - j\| > 4$)	0	3
Dihedral restraints	0	18
Lennard-Jones Energy (kJ mol^{-1})	-231± 23	-300 ± 20
**Structure statistics**		
Violations		
NOE violations >0.2 Å	0	0 ± 0.00375
Dihedral angle violations >2.0 Å	-	0 ± 0.18234
Ramachandran (%)		
Most favoured region	52.7	67.3
Additionally allowed region	33.1	25.8
Generously allowed region	9.6	3.1
Disallowed region	4.6	3.8
Average pairwise r.m.s. deviation (Å)*		
Heavy	5.140	2.610
Backbone	3.171	1.570

* Pairwise r.m.s. deviation was calculated over peptide residues 4-16.

Table S2. NMR refinement statistics for 20 structure ensembles of M2AH in solution and in presence of 100nm POPC:POPG:Cholesterol LUVs.

Supplemental Table 3

Residue	Assignment	Integral [abs]	% of max
F54	Hε1/2	1239392	34.6
F55	Hε1/2	3587120	100.0
H57	Hδ1/2	1764799	49.2
F48	Hδ1/2	1054350	29.4
F54/F55	Hδ1/2	2082138	58.0
Y52	Hε1/2	979446	27.3
Y52	Hδ1/2	865698	24.1
G58	Hα2/3	733069	20.4
F47/48/54/55	Hα	1581385	44.1
L59	Hδ1	864895	24.1
L59	Hδ2	837269	23.3
I51	Hδ	1879250	52.4

Table S3. STD values and percentage of the maximum transfer value for selected residues from M2AH in the presence of 100nm POPC:POPG:Cholesterol LUVs.
